# Supplementary material for: Cue-driven microbial cooperation and communication: evolving quorum sensing with honest signaling
Source: BMC Biol. 2024 Apr 2;22:73. doi: 10.1186/s12915-024-01857-6 (PMC10986144; doi:10.1186/s12915-024-01857-6)
Supplement: Supplementary file 1 — Additional file 1: Appendix 1, Mean-field model, Fig. S1; Appendix 2, Configuration-field model, Figs. S2-S5; Appendix 3. Individual-based lattice model, Fig. S6. [file 12915_2024_1857_MOESM1_ESM.pdf]

Supplementary material for

Cue-driven microbial cooperation and communication: evolving quorum sensing with honest signalling

Tamás Czárán[1], István Scheuring[1], István Zachar[1,2], Szabolcs Számadó[3,4]

1 HUN-REN, Institute of Evolution, Centre for Ecological Research, Konkoly-Thege Miklós út 29-33., H 1121 Budapest, Hungary
2 Department of Plant Systematics, Ecology and Theoretical Biology, Eötvös Lóránd University, H-1117 Budapest, Hungary
3 Department of Sociology and Communication, Budapest University of Technology and Economics, Egry J. u. 1, Budapest, H-1111, Hungary
4 HUN-REN, CSS-RECENS Lendület Research Group, Centre for Social Science, Tóth Kálmán u. 4, H-1097, Budapest, Hungary

This pdf file includes:

Supplementary text: the mean field model, the configuration field model and additional results for the lattice model
Figs S1 to S9
References

Appendix 1. The mean-field model

Let $x_1, x_2, x_3, x_4, x_5, x_6, x_7, x_8$ ($\sum x_i = 1$) denote the frequencies of the strategies of $La, Tr, Bo, Sm, Ne, Li, Cl, Vo$, respectively. $x_2+x_3+x_4+x_5 = X_C$ is the total concentration of cooperators, x_k is the cooperation threshold concentration and $X_C + x_3 + x_5 + x_6 + x_7 = X_S$ denotes the total concentration of signals. N is the neighborhood size, which is assumed to be large in this limit. By using the notation introduced in the main text and above the strategies have the following fitness in the mean field limit:

$$W_{La}(X_C) = W_0 - c_0\left[1 - b\theta(X_C)\right] \tag{1}$$

$$W_{Tr}(X_C) = W_0 - (c_0 + c)\left[1 - b\theta(X_C + 1/N)\right] \tag{2}$$

$$W_{Bo}(X_C) = W_0 - (c_0 + c + s)\left[1 - b\theta(X_C + 1/N)\right] \tag{3}$$

$$\begin{aligned} W_{Sm}(X_C, X_S) = W_0 - (c_0 + r + c)(1 - b)\theta(X_C + 1/N) - \\ \left[c_0 + r + c\theta_{S|1-C}(X_C + 1/N, X_S + 1/N)\right]\left[1 - \theta(X_C)\right] \end{aligned} \tag{4}$$

$$\begin{aligned} W_{Ne}(X_C, X_S) = W_0 - (c_0 + r + s + c)(1 - b)\theta(X_C + 1/N) - \\ \left[c_0 + r + s + c\theta_{S|1-C}(X_C + 1/N, X_S + 2/N)\right]\left[1 - \theta(X_C + 1/N)\right] \end{aligned} \tag{5}$$

$$W_{Li}(X_C) = W_0 - (c_0 + s)\left(1 - b\theta(X_C)\right), \tag{6}$$

$$W_{Cl}(X_C) = W_0 - (c_0 + s + r)\left(1 - b\theta(X_C)\right), \tag{7}$$

$$W_{Vo}(X_C) = W_0 - (c_0 + r)\left(1 - b\theta(X_C)\right), \tag{8}$$

where

$$\theta(X) = \begin{cases} 1, & \text{if } X \geq x_k \\ 0, & \text{otherwise} \end{cases}$$

,

$$\theta_{S|1-C}(X, Y) = \begin{cases} 1, & \text{if } X < x_k \text{ and } Y \geq x_k \\ 0, & \text{otherwise} \end{cases}$$

are step functions representing the effect of cooperation as functions of the cooperative strategies' concentrations and the signal concentrations. Since N is the neighborhood size, the focal strategy is taken into account with weight $1/N$ in the θ functions if it cooperates (eqs. 1-5) and/or as a signaler if it signals (eqs. 4,5). The Ne strategy issues an extra signal and it detects signals as well, so this strategy adds with weight $2/N$ to the signal concentration term when we compute

W_{Ne} (eq. 5). Naturally, as the the neighborhood size N tends to infinity, $\theta(X_C + 1/N) \to \theta(X_C)$; consequently, the La strategy attains the highest fitness regardless of the frequency distribution of the strategies. That is, La is always the winner of selection in this limit.

If N is finite - even if it is large - and b is sufficiently high compared to the costs c, s, r, then besides La, the inevitable winner of selection, also the cooperative strategies can be successful. Below we analyse this case. From the definitions of the strategies and the specific assumptions of the mean-field model (i.e., everyone detects the same amount of quorum signal and feels the same effect of cooperation across the habitat) it follows that

$$W_{La} > W_{Li}, W_{Cl}, W_{Vo}$$

and similarly

$$W_{Tr} > W_{Bo}, \quad W_{Sm} > W_{Ne},$$

regardless of the actual strategy distribution. Consequently, we need to consider the relations only among the La, Tr and Sm strategies, since all the others are certainly ousted by one of these three. Because all of these strategies are honest, $\theta_{S|1-C}(X, Y) = 0$, thus the fitnesses of these strategies in the mean-field model are simplified to:

$$\begin{aligned} W_{La}(X) &= W_0 - c_0 \left[1 - b\theta(X)\right] \\ W_{Tr}(X) &= W_0 - (c_0 + c) \left[1 - b\theta(X + 1/N)\right] \\ W_{Sm}(X) &= W_0 - \left[c_0 + r + c\right] (1 - b)\theta(X + 1/N) - (c_0 + r)[1 - \theta(X + 1/N)], \end{aligned} \tag{9}$$

where X is the joint concentration of the cooperative Tr and Sm strategies. For sake of simplicity the total concentration of strategies is rescaled to be between zero and 1.

Using standard assumptions of replicator dynamics (Hofbauer and Sigmund 1998) the system is represented by

$$\begin{aligned} \dot{x} &= (W_{La}(X) - \bar{W})x, \\ \dot{y} &= (W_{Tr}(X) - \bar{W})y, \\ \dot{z} &= (W_{Sm}(X) - \bar{W})z. \end{aligned} \tag{10}$$

where, x, y, z are the frequencies of the feasible La, Tr and Sm strategy triplet, and $\bar{W} = W_{La}x + W_{Tr}y + W_{Sm}z$ is the actual average fitness in the population. We studied the qualitative behavior of the above dynamical system by using the the fitness functions of (9).

Convergence and stability of the $x = 1$ state

Let us assume that the system is in the $x = 1, y = z = 0$ steady state. We are interested in the stability of this state. Assume that the invader Tr and Sm strategies emerge with frequencies y and z either together or separately. If their total frequency $X = y + z < x_k - 1/N$, then $\theta(X) = \theta(X + 1/N) = 0$ and consequently, $W_{La}(X) > W_{Tr}(X),\ W_{Sm}(x)$, so the invaders couldn't spread. Moreover, it follows from $y + z < x_k - 1/N$ that $x > 1 - x_k + 1/N$, consequently $\dot{x} > 0$ in(10) after invasion, therefore $\lim_{t\to\infty} x = 1$. Thus we have shown that the $x = 1, y = z = 0$ state is an asymptotically stable fixed point of the system if the total initial frequency of invaders satisfies $y + z < x_k - 1/N$.

Coexistence of La and Tr.

Now let us assume that $X = y+z \geq x_k - 1/N$. Then $\theta(X+1/N) = 1$ and thus $W_{Tr}(X) > W_{Sm}(X)$, at any parameter setting of the model. That is, the Sm strategy is always beaten by the Tr strategy. Thus we have to consider only the La/Tr subsystem at this initial condition, for which $z = 0$ and $y = 1 - x$ is the frequency of the Tr strategy. As we have shown above if $y < x_k - 1/N$ then $\dot{y} < 0$ in(10), so Tr is selected out. However, if $x_k - 1/N < y \leq x_k$ then $\theta(y) = 0$ while $\theta(y+1/N) = 1$. In this case, the dynamics depend on the parameters of

the model. If $b/c > 1/(c_0+c)$, then $W_{La}(y) < W_{Tr}(y)$ and, consequently, $\dot{y} > 0$, that is, y increases, so after a while y will be greater than x_k and then $\theta(y) = \theta(y + 1/N) = 1$. Thus, $W_{La}(y) > W_{Tr}(y)$, and, consequently, $\dot{y} < 0$, that is, y decreases again. We note here that since $W_{La}(x_k) < W_{Tr}(x_k)$, x_k is not a fixed point of the dynamics by definition, but $W_{La}(x_k + \varepsilon) > W_{Tr}(x_k + \varepsilon)$ for any small $\varepsilon > 0$.). This strange behavior of the system (no fixed point for the replicator dynamics but coexistence anyway) is the consequence that the θ step function used in the model is nonderivable at the point of the jump. Changing the step function to a continuous sigmoidal benefit function obliterates this singularity by transforming the average benefit of the strategies to a continuous, strictly monotonously increasing function of the strategy distribution. Then the stationary state analyzed above becomes the well known fixed point where $W_{Tr}(y*) = W_{La}(y*)$, with $y* \in (x_k - 1/N,\ x_k)$. Notice that any fluctuation decreasing y below $x_k - 1/N$ drives Tr extinct and fixates La (Fig. S1).

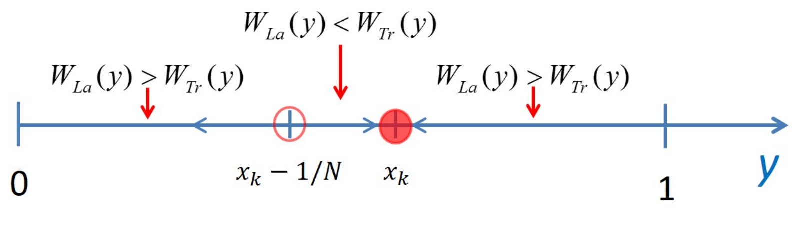

Figure S1 The qualitative dynamics of the La/Tr system. The $x_k - 1/N$ and x_k points separate three different dynamical behaviours.

As we have shown above the fitness of the Sm strategy is always lower than that of the Tr if $y \geq x_k - 1/N$; therefore, Sm can never invade a Tr/La coexistence.

Coexistence of La and Sm.

Considering the coexistence of Sm with La the situation is the same as before. The $(z = x_k, x = 1 - x_k)$ is a resting point around which the system fluctuates if $b > (r + c)/(c_0 + r + c)$. However, since $W_{Tr}(X) >$

$W_{Sm}(X)$ if $X = y + z \geq x_k - 1/N$, Tr always invades the La/Sm coalition and excludes Sm.

Naturally, if $b/c < 1/(c_0+c)$, then La always has a fitness higher than the alternative strategies Tr and Sm, thus La is the only fixed point of the replicator dynamics.

Appendix 2 The configuration field model

We assume that the interacting group around a focal individual is assembled by drawing $N-1$ individuals at random from the population. The probability of having at least k cooperators (or units of signal) in the group of N individuals thus assembled (including the focal individual) is the weighted average of having k, $k+1$,...N cooperators (or signal doses) within the group. Let us denote with $P_l(\vec{x})$ the average probability of having at least l cooperators among the $N-1$ group members if the frequency distribution of the 8 strategies is $(x_1, x_2, ..x_8) = \vec{x}$ in the population. Let $Q_{m|l}(\vec{x})$ be the average probability that the number of signal units is at least m while the number of cooperators is smaller than l in the group of $N-1$ neighbours if the global strategy frequencies are given by $\vec{x}$. Denote the threshold number of cooperators with k ($k \epsilon Z^+, k \leq N$). Using notations and arguments similar to those of the mean-field model we can determine the average fitnesses of the strategies as

$$W_{La}(\vec{x}) = W_0 - c_0[1 - bP_k(\vec{x})] \tag{11}$$

$$W_{Tr}(\vec{x}) = W_0 - (c_0 + c)[1 - bP_{k-1}(\vec{x})] \tag{12}$$

$$W_{Bo}(\vec{x}) = W_0 - (c_0 + s + c)[1 - bP_{k-1}(\vec{x})] \tag{13}$$

$$\begin{aligned} W_{Sm}(\vec{x}) = W_0 - (c_0 + r + c)(1 - b)P_{k-1}(\vec{x}) - \\ (c_0 + r + c)Q_{k-1|k-1}(\vec{x}) - \\ (c_0 + r)[1 - Q_{k-1|k-1}(\vec{x})][1 - P_{k-1}(\vec{x})] \end{aligned} \tag{14}$$

$$W_{Ne}(\vec{x}) = W_0 - (c_0 + r + s + c)(1-b)P_{k-1}(\vec{x}) - (c_0 + r + s + c)Q_{k-2|k-1}(\vec{x})] - (c_0 + r + s)[1 - Q_{k-2|k-1}(\vec{x})][1 - P_{k-1}(\vec{x})] \tag{15}$$

$$W_{Li}(\vec{x}) = W_0 - (c_0 + s)\left[1 - bP_k(\vec{x})\right], \tag{16}$$

$$W_{Cl}(\vec{x}) = W_0 - (c_0 + s + r)\left[1 - bP_k(\vec{x})\right], \tag{17}$$

$$W_{Vo}(\vec{x}) = W_0 - (c_0 + r)\left[1 - bP_k(\vec{x})\right], \tag{18}$$

Like in the the mean field model, it can be shown that $W_{La} > W_{Li}, W_{Cl}, W_{Vo}$ and $W_{Tr} > W_{Bo}$, $W_{Sm} > W_{Ne}$, regardless of the strategy distribution. Thus again only the La, Tr or Sm strategies can be present in any equilibrium state. Assuming random selection of individuals into the interacting groups the fitnesses of these three strategies are

$$\begin{aligned} W_{La}(x,y,z) &= W_0 - c_0[1 - bP_k(x,y,z)] \\ W_{Tr}(x,y,z) &= W_0 - (c_0 + c)[1 - bP_{k-1}(\vec{x})] \\ W_{Sm}(x,y,z) &= W_0 - (c_0 + r + c)(1-b)P_{k-1}(x,y,z) - (c_0 + r)[1 - P_{k-1}(x,y,z)], \end{aligned}$$

with the probabilities computed as

$$P_k(x,y,z) = \sum_{i+j\geq k} f_{i,j}(N-1)x^{N-1-i-j}y^i z^j,$$

$$P_{k-1}(x,y,z) = \sum_{i+j\geq k-1} f_{i,j}(N-1)x^{N-1-i-j}y^i z^j.$$

$f_{i,j}(N-1)$ is the corresponding trinomial coefficient and x, y, z are the frequencies of the La, Tr and Sm strategies, respectively. Since there are no non-cooperating signaling strategies in this subsystem, $Q_{k-1|k}(x,y,z) = 0$ at any x, y, z frequency. We consider the same type of replicator equations as above (10) and study this dynamical system qualitatively.

La **is a locally asymptotically stable state of the replicator dynamics.**

We consider the $(x = 1, y = z = 0)$ state and show that the rare invader Tr or Sm strategies have always lower fitness than the resident La strategy. Let us assume first that the invaders follow the Tr strategy with frequency $y << 1$. The success of invasion is determined by the sign of fitness difference of the resident and the invader. After some elementary calculations the fitness difference of La and Tr is determined by

$$W_{La}-W_{Tr} = c[1-bP_{k-1}(1-y,y,0)]+c_0 b[P_k(1-y,y,0)-P_{k-1}(1-y,y,0)].$$

Since $\lim_{y\to 0} P_{k-1}(1-y,y,0) = \lim_{y\to 0} P_k(1-y,y,0) = 0$, the fitness difference tends to $c > 0$ if $y \to 0$. Thus $Sign\{W_{La} - W_{Tr}\} > 0$, that is, the fitness of the invader Tr is always smaller than that of the resident La if the invader is rare. Strategy Tr can not spread if $y << 1$.

Similarly, La is resistant against the invasion of rare Sm $(z << 1)$. The fitness difference of La and Sm is

$$(c_0 + r + c)(1-b)P_{k-1}(1-z,0,z)+(c_0 + r)[1 - P_{k-1}(1-z,0,z)]- \\ c_0[1 - bP_k(1-z,0,z)] \tag{19}$$

which, for the same reason as above, tends to $r > 0$ as $z \to 0$. Strategy Sm can not spread if $z << 1$.

Neither Tr **nor** Sm **are stable against the invasion of** La.

Consider again the fitness differences of La and Tr or La and Sm, but at the $y \approx 1$ and $1-y \approx 0$ or at $z \approx 1$ and $1-z \approx 0$ initial values, respectively. Since $P_l(0,1,0) = 1$, for $l \in Z^+$; $l \leq N$,

$$\lim_{y\to 1} W_{La}(1-y,y,0) - W_{Tr}(1-y,y,0)\} = c\,[1-b]\,,$$
$$\lim_{z\to 1} W_{La}(1-z,0,z) - W_{Sm}(1-z,0,z) = (r+c)\,[1-b]\,.$$

Since these limits are always positive, La always has higher fitness than Tr or Sm if La is rare, that is, La successfully invades both cooperative strategies.

The stable coexistence of La and Tr

We are look for frequencies y where

$$W_{La}(1-y,y,0) = W_{Tr}(1-y,y,0), \tag{20}$$

i.e., where La and tr are at equilibrium. Substituting the fitness values and rearranging (20) yields the condition of fitness equilibrium as

$$\frac{c}{b} = cP_{k-1}(1-y,y,0) + c_0 F_k(y) = H_1(y), \tag{21}$$

where $F_k(y) = f_{k-1}(N-1)(1-y)^{N-k}y^{k-1}$. It is easy to see that $H_1(0) = 0$ and $H_1(1) = c$. Since $0 < b < 1$, $H_1(0), H_1(1) < c/b$.

Now we show that $H_1(y)$ has only one maximum ($Max\{H_1(y)\} = H_1(y^*)$) in $y \in [0,\ 1]$. We apply that $F_k(y)$ has a single maximum (see e.g. Archetti and Scheuring 2010) in $y \in [0,\ 1]$. Further, it can be shown that $P_{k-1}(1-y,y,0)$ is a monotonously increasing function with $P'_{k-1}(1,0,0) = P'_{k-1}(0,1,0) = 0$ and with an inflexion point at a $y_k \in (0,\ 1)$. To prove this let us observe that $P_{k-1}(1-y,y,0) = \sum_{i=0}^{N-1}\theta((i+1)/(N-1))f_i(N-1)y^i(1-y)^{N-1-i}$ where $\theta(x)$ is a step function defined as $\theta(x)$=0 if $x < k$ and $\theta(x)$=1 if $x \geq k$. Actually, $P_{k-1}(1-y,y,0)$ is the Bernstein polynomial $B_{N-1}(\theta; y)$ of $\theta(y)$ (Phillips 2003). We introduce the function

$$\sigma_\alpha(y) = \frac{1}{1+e^{-\alpha(y-(k-1)/(N-1))}},\ y \in [0,\ 1], \tag{22}$$

and define

$$T_\alpha(y) = \frac{\sigma_\alpha(y) - \sigma_\alpha(0)}{\sigma_\alpha(1) - \sigma_\alpha(0)}, y \in [0,\ 1]. \tag{23}$$

For every $\varepsilon_1 > 0$ there is an α_c that for every $\alpha > \alpha_c$, $\|\theta(y) - T_\alpha(y)\| < \varepsilon_1$ for every $y \in [0,\ 1]$. That is, $T_\alpha(y)$ is an arbitrarily good continuous approximation of the step function $\theta(y)$. Since the Bernstein operator

is monotonous (Phillips 2003),

$$\|B_{N-1}(\theta; y) - B_{N-1}(T_\alpha; y)\| < \varepsilon_2 \tag{24}$$

remains valid for every $\varepsilon_2 > 0$ if α is sufficiently large. $B_{N-1}(T_\alpha; y)$ is an arbitrary good approximation of $P_{k-1}(y, 1-y, 0)$. $T_\alpha(y)$ is a continuous and differentiable function, thus if it is monotonous then its Bernstein polynomial is a monotonous function as well (Phillips 2003). Consequently, $B_{N-1}(T_\alpha; y)$ is monotonously increasing, and because of (24) $P_{k-1}(y, 1-y, 0)$ is a monotonously increasing function as well. Further, the Bernstien operator does not change the sign of the derivatives of a function. That is, since $T'_\alpha(y) > 0$, it means that $B'_{N-1}(T_\alpha; y) > 0$, and consequently $P'_{k-1}(1-y, y, 0) > 0$. Similarly, since the sign of $T''_\alpha(y)$ changes from positive to negative at the inflexion point, so do $B''_{N-1}(T_\alpha; y)$ and $P''_{k-1}(1-y, y, 0)$ (Phillips 2003).

$H_1(y)$ has maximum (or minimum) points where $H'_1(y) = 0$, that is, where

$$cP'_{k-1}(1-y, y, 0) = -c_0 F'_k(y). \tag{25}$$

It is easy to show that $F_k(y)$ is a single-humped function with a maximum point $\hat{y} \in (0,\ 1)$. Similarly, it can be shown that $P'_{k-1}(1-y, y, 0)$ is also a single-humped function with one maximum value in the $[0,\ 1]$ range. Since $F'_k(y) > 0$ in $y \in [0, \hat{y})$, and $F_k(0) = P_{k-1}(1, 0, 0) = 0$, eq. (25) has exactly one solution if

$$\lim_{y \to 1} cP'_{k-1}(1-y, y, 0) < \lim_{y \to 1} -c_0 F'_k(y). \tag{26}$$

Figure (S2) visualizes these two functions by a numerical example. After derivation of the functions and rearranging the above relation we get

$$\lim_{y \to 1} c \sum_{i \geq k} f_i (N-1) \frac{y^{i-1}}{y^{k-2}} \frac{(1-y)^{N-2-i}}{(1-y)^{N-k-1}} (i - (N-1)y) <$$
$$\lim_{y \to 1} -(c_0 + c) f_{k-1} (N-1) \left((k-1) - (N-1)y\right).$$

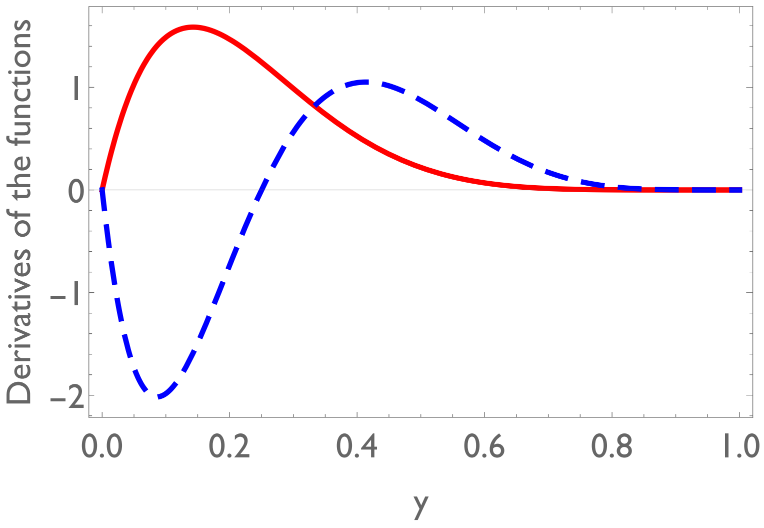

Figure S2 The shape of $cP'_{k-1}(1-y,y,0)$ (red) and $-c_0F'_k(y)$ (dashed, blue) functions if $H_1(y)$ has one maximum. The intersection of these functions determines the maximum point of $H_1(y)$. $c = 0.5$, $c_0 = 1$, $k = 3$, $N = 9$, in this example.

The left hand side of the relation tends to $-\infty$ and the right hand side tends to $(N-k-1)(c+c_0) > 0$, so relation (26) is satisfied.

Thus we have proven that $H_1(y)$ has only one maximum at y^* in $y \in [0 \quad 1]$. If $H_1(y^*) > c/b$ then there are two solutions $y_u < y^* < y_s$ of eq. (21), where y_u is the unstable and y_s is the stable equilibrium of the La-Tr coexistence. (The stability can be studied by computing the signs of the fitness differences above and below the equilibrium y_u and y_s frequencies). Contrary, if $H_1(y^*) < c/b$ then $W_{La} > W_{Tr}$ for every $y \in [0,1]$, therefore the coexistence of these strategies is not possible (Archetti and Scheuring 2010). In summary, there is at most one stable polymorphic equilibrium (y_s) of La and Tr strategies, and an unstable one (y_u) that separates the trajectories. If $y < y_u$ initially then the system evolves to the pure La strategy $(y = 0)$, while if $y > y_u$ then it converges to the state of coexistence (y_s).

It follows from (21) that there is a critical $(c/b)^*$ below which $H_1(y^*) < (c/b)^*$ for every c_0, that is, when La always outcompetes Tr, and thus the La strategy remains the only stable state of the dynamics. Therefore besides the fact that La strategy that is always locally stable , the

unstable and stable fixed points (y_u, y_s) emerge in pair by a blue sky bifurcation (Fig S3)

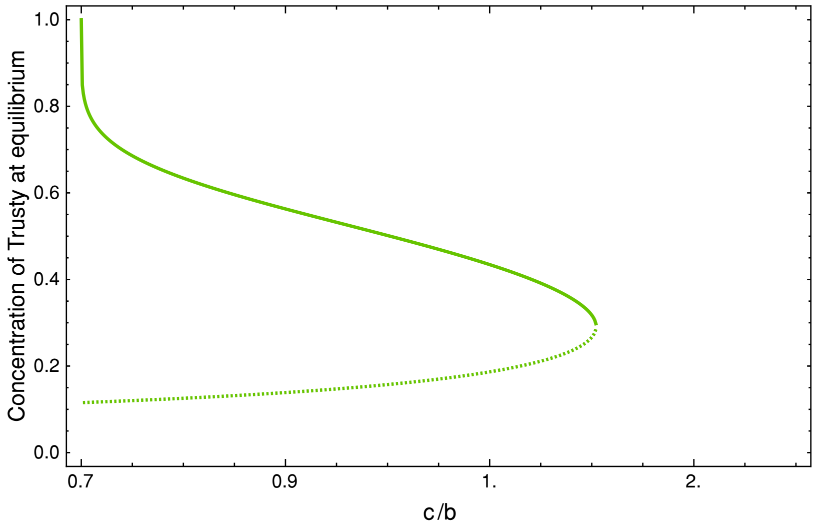

Figure S3 The bifurcation diagram of the $La - Tr$ subsystem. The stable (solid line) and unstable (dashed line) fixed points appear in pair below a critical c/b value. Other parameters $k = 3,\ N = 9,\ c_0 = 3, w_0 = 1/10$ in this example.

The stable coexistence of La and Sm.

We perform essentially the same analysis as before; however, in this case equation (21) is modified to

$$\frac{r}{b} = (r + c - c/b)P_{k-1}(1 - z, 0, z) + c_0 F_k(z) = H_2(z) \qquad (27)$$

as the condition for the coexistence of La and Sm. As before, it can be shown easily that $H_2(0) = 0$ and $H_2(1) = r + c - c/b$, which are both smaller than r/b if $0 < b < 1$. Since $H_2(z)$ is the sum of the same polynomials as $H_1(y)$, we can use the same method as before to show that the $H_2'(z) = 0$ equation has at most one solution in $z \in (0,\ 1)$, and consequently, $H_2(z) = \frac{r}{b}$ has two solutions: z_u and z_s if $Max\{H_2(z)\} > \frac{r}{b}$ in $z \in (0,\ 1)$. There is no solution if $Max\{H_2(z)\} < \frac{r}{b}$. (In the marginal case when $Max\{H_2(z)\} = \frac{r}{b}$, $z_u = z_s$.) The only difference is that $\lim_{z\to 0} H_2'(z)$ can be negative because $r + c - c/b$ can be negative as well.

The following cases are possible:

- If $\lim_{z\to 0} H_2'(z) > 0$ then the situation is the same as before, so there is only one $z^* \in (0,\ 1)$ where $H_2'(z^*) = 0$. This is the point where $H_2(z)$ is maximal (Fig. S4). If $H_2(z^*) > r/b$, then there is an unstable and a stable fixed point of the replicator dynamics of La and Sm in $z \in (0,\ 1)$. Contrary, if $H_2(z^*) \leq r/b$, then $W_{La} > W_{Sm}$ for every $z \in (0,\ 1)$, and thus La beats Sm.

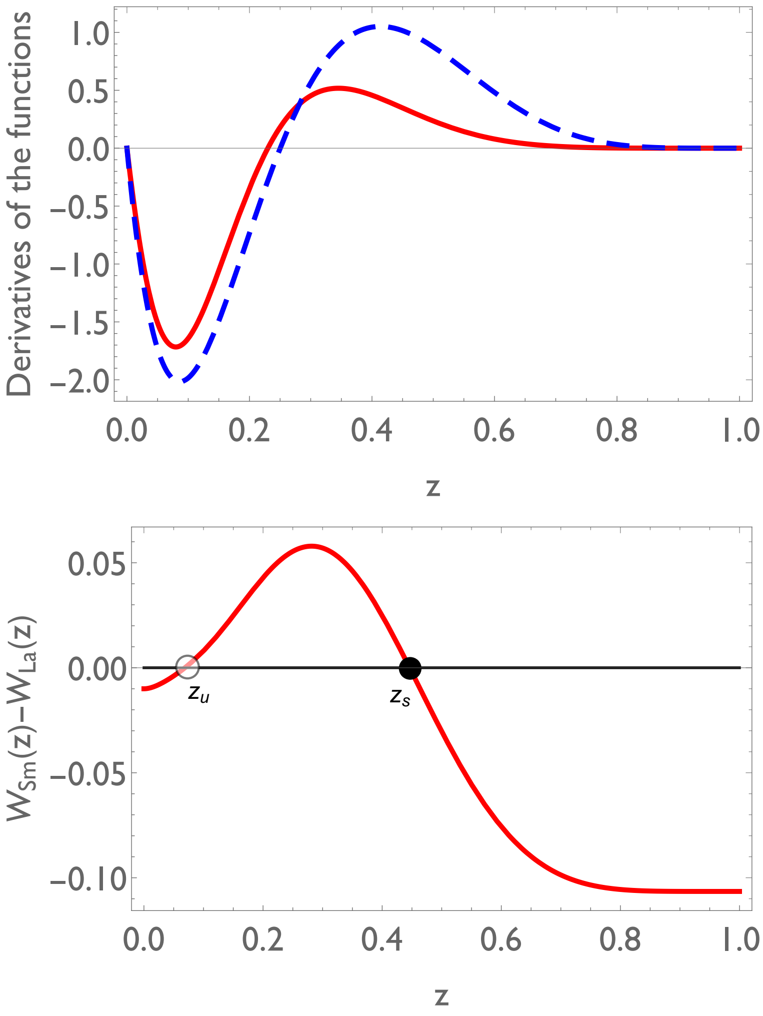

Figure S4 A case when $H_2'(z)$ is zero at a single $z_{max} \in [0,\ 1]$. **up:** $(r + c - c/b)\, P_{K-1}'(1 - z, 0, z)$ (red), (-$c_0 F_k'(z)$ dashed, blue). **bottom:** The fitness difference of Sm and La in function of z. The dynamics has an unstable (z_u) and a stable (z_s) fixed point. $N = 9, k = 3, c_0 = 1, c = 0.7, b = 0.85, r = 0.01$

- If $\lim_{z\to 0} H_2'(z) < 0$ then, since $P_{k-1}'(1 - z, 0, z) \geq 0$ and $F_k'(z)$ changes the sign exactly once, there are two possibilities: either $H_2'(z) < 0$ for every $z \in [0,\ 1]$ or $H_2'(z)$ changes sign two times

in $z \in [0,\ 1]$ (Fig. S5). In the first case $W_{La} > W_{Sm}$ for every

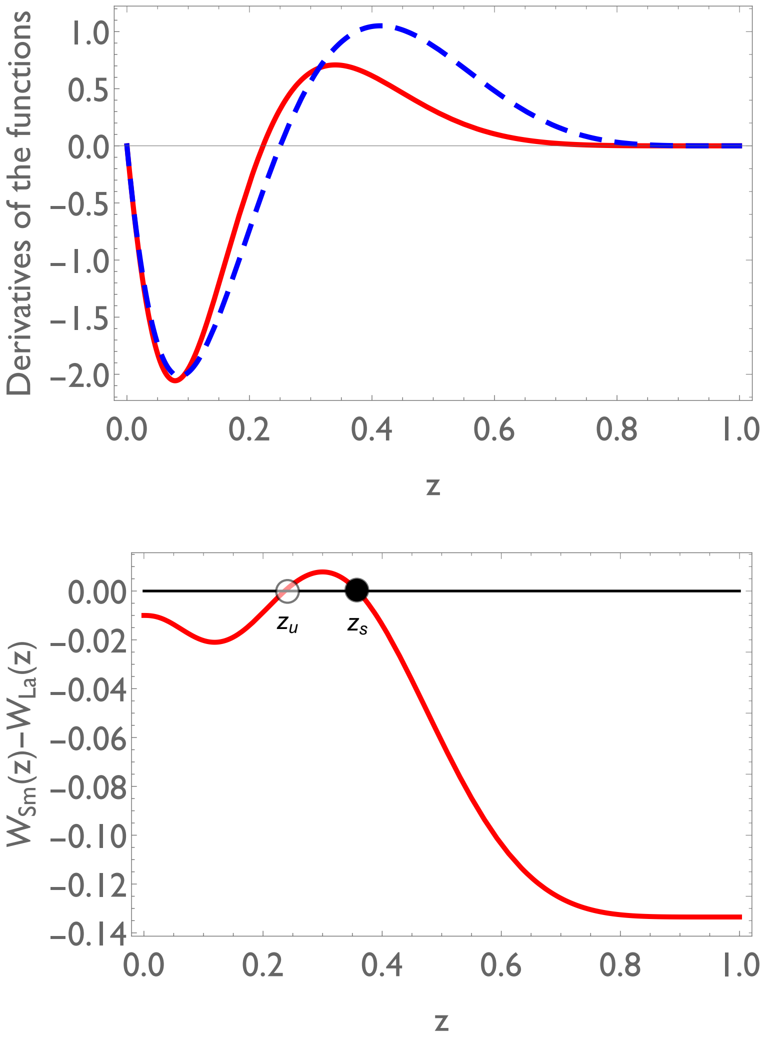

Figure S5 A case when $H_2'(z)$ becomes zero two times; i.e., there is a local minimum and maximum point of this function.**up:** $(r + c - c/b)\, P_{k-1}'(1 - z, 0, z)$ (red) -$c_0 F_k'(z)$ (dashed, blue). **bottom:** The fitness differences in function of z. Again there are z_u and z_s unstable and stable fixed points of the dynamics. $N = 9, k = 3, c_0 = 1, c = 0.88, b = 0.85, r = 0.01$

$z \in [0,\ 1]$, so La wins the selection (Fig. S6). In the second case $H_2(z)$ has a minimum value at z^{**} and a maximum value at z^*. Again if $H_2(z^*) > r/b$, then there is an unstable (z_u) and a stable (z_s) fixed point of the dynamics of the La and Sm subsystem, otherwise La wins the competition. Thus the z_u z_s fixed points emerge by blue sky bifurcation as the r/b parameter changes (Fig. S7).

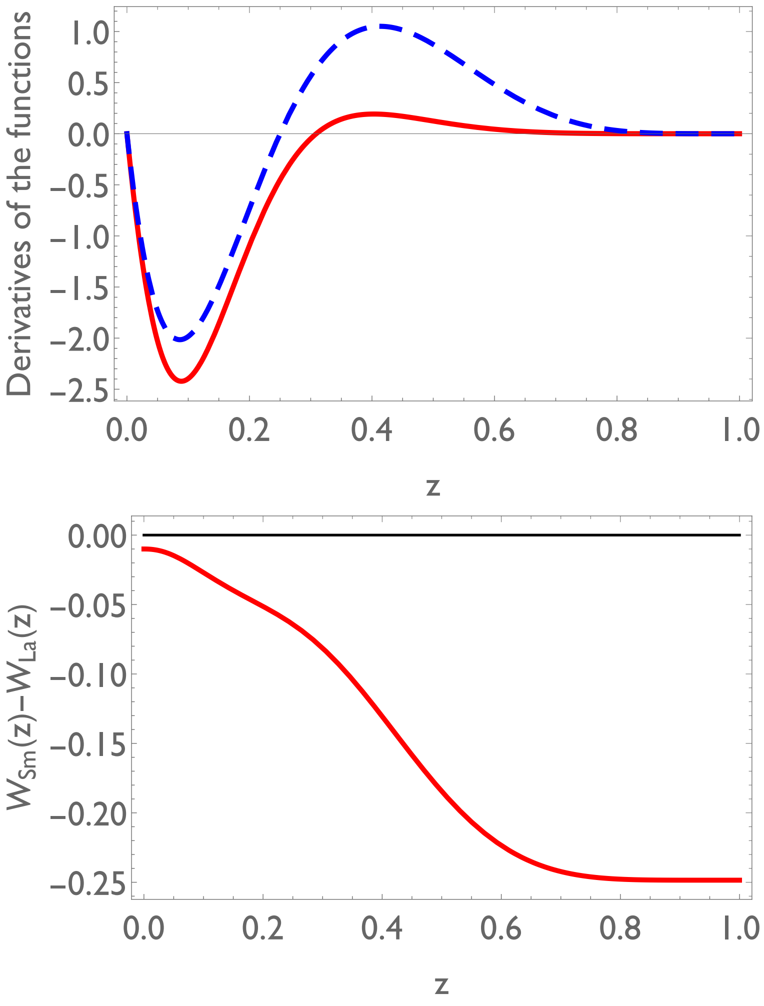

Figure S6 A case when $H_2'(z) < 0$ and thus La wins over Sm. **up:** $(r + c - c/b)\, P_{k-1}'(1 - z, 0, z)$ (red) $-c_0 F_k'(z)$ (dashed, blue). **bottom:** The fitness differences in function of z. $N = 9, k = 3, c_0 = 1, c = 0.7, b = 0.65, r = 0.01$

Invasion of two coexistent strategies by the third one

Let us assume that there is a stable polymorph steady state of La and Tr. The rare Sm can invade this state if its fitness is higher than the fitness of La or Tr in the equilibrium. Let y_s denote the frequency of Tr when Tr and La are in stable equilibrium; thus the fitness of the rare invading Sm is

$$W_0-(c_0+r+c)[1-b]P_{k-1}(1-y_s,y_s,0)]-(c_0+r)[1-P_{K-1}(1-y_s,y_s,0)].$$

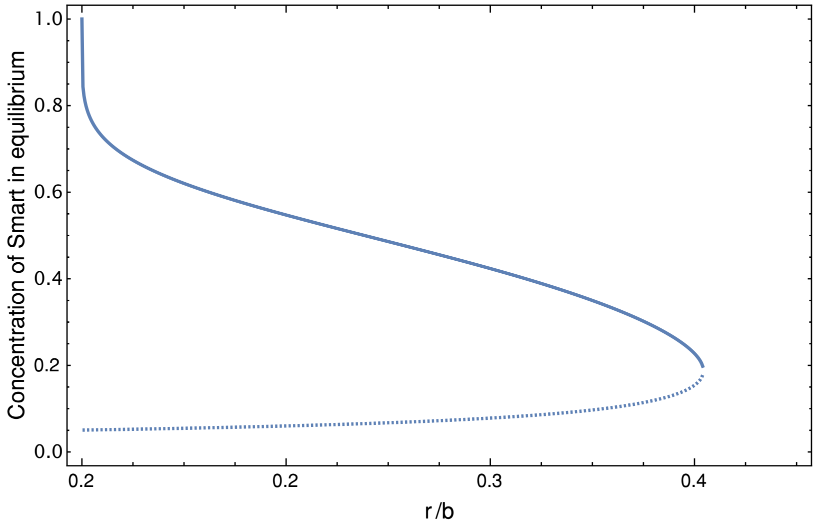

Figure S7 The bifurcation diagram of the $La - Sm$ subsystem. The stable (solid line) and unstable (dashed line) fixed points appear in pair below a critical r/b value. Other parameters $k = 3,\ N = 9,\ c_0 = 3, w_0 = 1/10,\ c = 4/5,\ k = 3,\ N = 9,$ in this example.

Since $P_l(1-y, y, 0) = P_l(1-z, 0, z)$ if $z = y$, for every $l = 1, 2, N-1$ the above fitness function can be rewritten as $W_0 - [c_0 + r + c][1 - b]P_{k-1}(1-y_s, 0, y_s) - [c_0 + r][1 - P_{k-1}(1-y_s, 0, y_s)]$ as the fitness of the rare invading Sm at the stable polymorph state of Tr and La. Sm can invade this polymorph Tr, La state only if there is a z_s frequency at which La and Sm are in stable coexistence, otherwise the fitness of Sm is always smaller than the fitness of La. Let z_u and z_s be the frequency of Sm where La and Sm are in unstable and stable equilibrium. Assume that $y_s \in (z_u,\ z_s)$. Since $W_{Sm}(z) > W_{La}(z)$ for every $z \in (z_u,\ z_s)$, $W_{Sm}(y_s) > W_{La}(y_s) = W_{Tr}(y_s)$, that is, Sm can spread when rare in the polymorph state of La and Tr. Since $y_s < z_s$, $W_{Tr}(z_s) < W_{La}(z_s) = W_{Sm}(z_s)$ follows; that is, Tr can't spread at the Sm-La equilibrium.

Using similar arguments it can be shown that if $z_s \in (y_u,\ y_s)$ the stable polymorph state of Tr and La resists the invasion of the rare Sm strategy, and the polymorph state of Sm and La is unstable against the invasion of Tr.

The remaining possibilities are that either $z_s < y_u$ or $y_s < z_u$. Then the system is bistable: neither Tr nor Sm can invade when they are rare. The existence of these two stable fixed points requires the existence of at

least one inner (where the three strategies coexist) unstable fixed point. However, we will see later that the coexistence of the three strategies is not possible. Therefore, this bistability is also not possible. Combining these invasion rules and bifurcation diagrams of $La - Tr$ and $La - Sm$ subsystems, the La strategy can form a stable state either with Sm or with Tr, depending on the model parameters (Fig. S8).

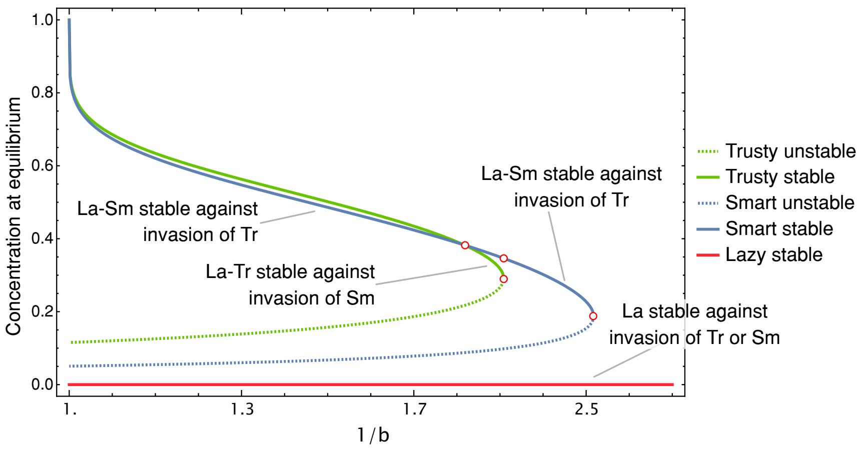

Figure S8 The bifurcation diagrams of $La - Sm$ and $La - Tr$ subsystems as a function of $1/b$. The invasion stable subsystems are determined by the relative values of stable fixed points in the subsystems. Other parameters $k = 3$, $N = 9$, $c_0 = 3, w_0 = 1/10$, $c = 4/5$, $r = 1/6$, $k = 3$, $N = 9$, in this example.

Coexistence of Sm and Tr is not possible.

The condition for coexistence is that

$$\frac{c - r}{c - rb} = P_{k-1}(0, y, 1 - y). \tag{28}$$

Since $P_k(0, y, 1 - y) = 1$ for every $y \in [0,\ 1]$ and the left-hand side is smaller than 1, the above equation can not be satisfied, except in the trivial case of $r = 0$. Otherwise, Sm has a smaller fitness than Tr so it is competed out.

Coexistence of the three strategies is not possible.

For the coexistence of La, Tr and Sm it is necessary that there is a $(\hat{x} > 0, \hat{y} > 0, \hat{z} > 0)$ where $W_{Tr} = W_{Sm} = W_{La}$. W_{Tr} and W_{Sm} are

equal if

$$(c - r)/(c - rb) = P_{k-1}(\hat{x}, \hat{y}, \hat{z}) \tag{29}$$

where $\hat{z} = 1 - \hat{x} - \hat{y}$.

It is clear again, that (29) can be valid only if $r = 0$ and if $\hat{x} = 0$, which excludes the possibility of coexistence of the all three strategies.

Appendix 3. Lattice model

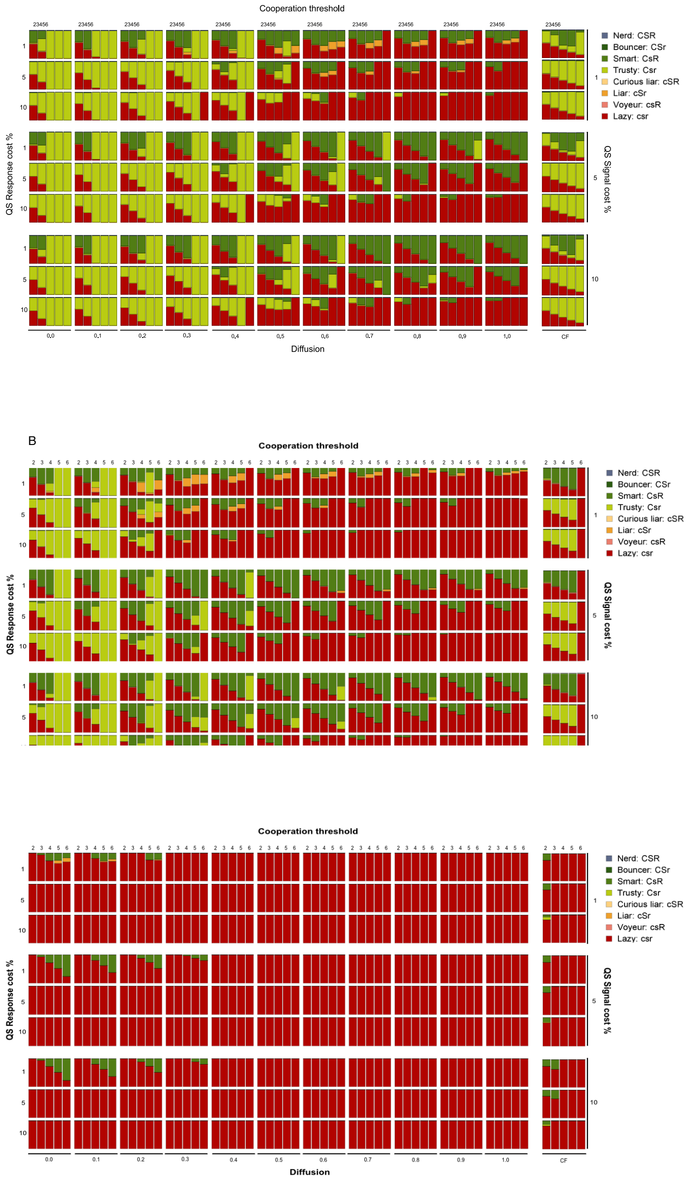

Figure S9 Genotype distributions in steady-state populations of the 8-strategy lattice model and the CF model across the feasible ranges of cooperation threshold (k), QS signal cost (s), QS signal response cost (r) and agent mobility due to diffusion (D), with fixed parameters $N = 9, c_0 = 1.0$ in all cases. The functional mutation rate for all strategies is $\rho = 10^{-4}$. A: $c = 0.2, b = 0.8$; B: $c = 0.3, b = 0.8$; C: $c = 0.3, b = 0.5$.

References

Archetti, M. and Scheuring, I. (2010). Coexistence of cooperation and defection in public goods games *Evolution*, **65:** 1140-1148.

Hofbauer, J. and Sigmund, K. (1998) *Evolutionary Games and Population Dynamics*. Cambridge University Press, Cambridge

Phillips, GM. (2003). *Interpolation and Approximation by Polynomials*, Springer New York, NY
